# Virology Outcomes of Tenofovir-lamivudine-dolutegravir in Treatment-naïve and Virologically Suppressed Individuals Switching From an NNRTI-based Regimen: An Observational Analysis at 13 Sites

**DOI:** 10.1093/ofid/ofaf270

**Published:** 2025-05-02

**Authors:** Cissy Kityo, Caitlyn McCarthy, Serena P Koenig, Michael D Hughes, Carole L Wallis, Isaac Tsikhutsu, Cornelius Munyanga, Noluthando Mwelase, Marije Van Schalkwyk, Jean Bernard Marc, Kelvin Mponda, Rodney Dawson, Fatma F Some, Lerato Mohapi, Yvetot Joseph, Urvi M Parikh, N Sarita Shah, Yukari C Manabe, Catherine Godfrey, Elizabeth Woolley, John W Mellors, Charles Flexner, ACTG A5381/Hakim Study Team, Marie Jude Jean Louis, Daphie Jean François, Vanessa Rouzier, Damocles Patrice Severe, Mina C Hosseinipour, Elliot Raizes, Deborah Langat, Mohammed Rassool, Vuyokazi S Jezile, Thando Mwelase, Abraham Siika, Viola Kirui, Rosie Mngqibisa, Penelope Madlala, Petronella Casey, Wadzanai Samaneka, Yeukai Musodza, Nadia Magengo, Suri Moonsamy, Mulinda Nyirenda, Francis Kanyike, Lynne Cornelissen, Lindee Ganger

**Affiliations:** Joint Clinical Research Centre (JCRC)/Kampala CRS, Kampala, Uganda; Center for Biostatistics in AIDS Research in the Department of Biostatistics, Harvard T.H. Chan School of Public Health, Boston, Massachusetts, USA; Les Centres GHESKIO Clinical Research Site (GHESKIO-INLR) CRS, Port-au-Prince, Haiti; Center for Biostatistics in AIDS Research in the Department of Biostatistics, Harvard T.H. Chan School of Public Health, Boston, Massachusetts, USA; Innovative Clinical Laboratories, Johannesburg, South Africa; Kenya Medical Research Institute/Walter Reed Project Clinical Research Center (KEMRI/WRP) CRS, Kericho, Kenya; Malawi Clinical Research Site (CRS), Lilongwe, Malawi; University of the Witwatersrand Helen Joseph (WITS HJH) CRS, Johannesburg, South Africa; Family Centre for Research with Ubuntu (FAMCRU) CRS, Stellenbosch University, Cape Town, South Africa; Les Centres GHESKIO Clinical Research Site (GHESKIO-INLR) CRS, Port-au-Prince, Haiti; Blantyre Clinical Research Site, Johns Hopkins Research Project, Blantyre, Malawi; University of Cape Town Lung Institute, Cape Town, South Africa; Moi University Clinical Research Center (MUCRC) CRS, Eldoret, Kenya; Perinatal HIV Research Unit (PHRU), University of the Witwatersrand, Johannesburg, South Africa; GHESKIO Institute of Infectious Diseases and Reproductive Health (GHESKIO-IMIS), Port-au-Prince, Haiti; University of Pittsburgh Department of Medicine, Division of Infectious Diseases, Pittsburgh, Pennsylvania, USA; Emory Rollins School of Public Health, Emory University, Atlanta, Georgia, USA; The Johns Hopkins University School of Medicine, Baltimore, Maryland, USA; Walter Reed Army Institute of Research, Department of Defense, Kampala, Uganda; DLH Corporation, Silver Spring, Maryland, USA; University of Pittsburgh Department of Medicine, Division of Infectious Diseases, Pittsburgh, Pennsylvania, USA; The Johns Hopkins University School of Medicine, Baltimore, Maryland, USA

**Keywords:** Africa region, ART, LMIC, observational prospective study, viral suppression

## Abstract

**Background:**

Tenofovir/lamivudine/dolutegravir (TLD) is widely prescribed worldwide. We report virologic and resistance outcomes for patients initiating or switching to TLD.

**Methods:**

A prospective observational study was performed at 13 AIDS Clinical Trials Group sites in 6 President's Emergency Plan for AIDS Relief-supported countries coincident with TLD rollout. This report includes results from 2 groups: group 1 (Gp1) were virally suppressed on nonnucleoside reverse transcriptase inhibitor-based antiretroviral therapy (ART) and group 2 (Gp2) were ART-naïve at TLD initiation. The primary objective was to estimate the proportions of participants with HIV-1 RNA ≤1000 copies/mL and frequency of dolutegravir resistance mutations 6 months after TLD initiation.

**Results:**

From October 2019 through July 2022, we enrolled 425 participants in Gp1 and 179 in Gp2. Two in Gp1 (0.5%) and 3 in Gp2 (1.7%) discontinued TLD by 6 months due to adverse events considered related to TLD (n = 4) and participant decision (n = 1). Ninety-three percent of participants in Gp1 and 92% in Gp2 who were still on TLD had a 6-month plasma HIV-1 RNA. Plasma HIV-1 RNA ≤1000, ≤ 200, and <50 copies/mL was achieved in 99%, 98%, and 96% in Gp1 and in 90%, 87%, and 85% in Gp2, respectively. A new integrase mutation (T97A/T) was observed in 1 participant in Gp1 and none in Gp2.

**Conclusions:**

TLD was well tolerated and achieved or maintained viral suppression (≤1000 copies/mL) in 90% of ART-naïve and 99% of participants with preswitch viral suppression. An emerging integrase strand transfer inhibitor mutation of uncertain significance was detected in only 1 participant. These data support early tolerability, virologic efficacy, and rare integrase strand transfer inhibitor resistance emergence with TLD transition or initiation in programmatic settings.

Dolutegravir-based antiretroviral therapy (ART) regimens have fewer drug–drug interactions, improved tolerability and greater potency, compared with previously recommended regimens [1–8] and a high genetic barrier to resistance [9, 10]. Systematic reviews have demonstrated the comparative efficacy and tolerability of integrase strand transfer inhibitors (INSTIs), particularly dolutegravir (DTG), in achieving and maintaining viral load (VL) suppression up to 96 weeks without treatment-emergent resistance [11–13]. In addition, epidemiological modeling using African data has shown that scaling up DTG-based regimens could curb nonnucleoside reverse transcriptase inhibitor (NNRTI)-resistance, reducing pretreatment drug resistance from about 52% (without DTG) to 10% (with universal DTG use) by 2040 [14].

The fixed-dose combination of tenofovir disoproxil fumarate, lamivudine, and DTG (TLD), is an optimal regimen for use in low- and middle-income countries. The efficacy, cost, and favorable resistance characteristics have led to changes in the World Health Organization [15, 16] and the President's Emergency Plan for AIDS Relief (PEPFAR) [17] guidelines for the use of ART for persons with HIV (PWH) to INSTI-based regimens for treatment-naïve and treatment-experienced patients, especially in settings where resistance testing prior to ART initiation may not be feasible [15, 17, 18]. These guidelines, together with licensing and pricing agreements, have further catalyzed the global roll-out and access to generic DTG-based therapy, especially for low- and middle-income countries, where about 100 countries have implemented this transition.

Before the start of this study, there was a paucity of data on the risks, benefits, and emergence of DTG resistance in TLD rollout programs where viral load measurement does not always happen expeditiously and drug resistance testing is not widely used to guide ART management [17]. To address this gap, the Advancing Clinical Therapeutics Globally (ACTG) protocol A5381 (The Hakim Study) was designed to systematically track clinical and virologic outcomes among PWH who were initiating or transitioning to TLD while receiving care through a PEPFAR-supported program.

## METHODS

### Study Design and Participants

The Hakim Study was a prospective, longitudinal, observational cohort study to assess the therapeutic efficacy and emergence of HIV drug resistance following initiation or switch to TLD [19]. The primary objective was to estimate, 6 months after initiation of TLD, the proportion of participants with HIV-1 RNA ≤1000 copies/mL, and the proportion with new DTG resistance mutations. Group 1 (Gp1) includes participants on NNRTI-based ART for at least 6 months with HIV-1 RNA ≤1000 copies/mL before switch to TLD and group 2 (Gp2) includes participants initiating ART with TLD.

Participants were recruited and followed at 13 PEPFAR-supported clinical sites in 6 countries (South Africa, Malawi, Zimbabwe, Uganda, Kenya, and Haiti). To be eligible for participation, participants needed to be ≥10 years old with documented HIV-1 and receiving care at a PEPFAR-supported site. Additionally, participants enrolled into Gp1 had to have been taking NNRTI-containing first-line ART for at least 6 consecutive months prior to study entry with HIV-1 RNA ≤1000 copies/mL before switch to TLD. Participants in group 2 were expected to be ART-naïve (although women who received ART only during pregnancy and/or breastfeeding for prevention of mother-to-child transmission, but who had not taken any ART for at least 6 calendar months immediately prior to study entry, were allowed).

### Ethical Considerations and Patient Consent Statement

The trial was approved by local ethics committees and national regulatory agencies in the respective countries. All participants provided written informed consent or assent and parental/guardian informed consent if they were younger than age 18 years.

### Procedures

To the extent practically possible, all individuals on NNRTI-based ART or starting TLD at selected sites were approached for screening and enrollment on the study after signing informed consent. Enrolled participants were managed according to local standards of care, and preferably in accordance with current World Health Organization Guidelines for HIV treatment and monitoring [15]. Eligible participants initiated or switched to TLD provided by a PEPFAR-supported program within 7 days after enrollment.

Visits took place at study entry and at months 3 and 6. Targeted clinical events (including AIDS-defining conditions, fractures, coronary heart disease, cancer, death, depression, diabetes, immune reconstitution inflammatory syndrome, suicidal ideation, suicide attempt, tuberculosis, and HIV-associated nephropathy) and adverse events leading to TLD discontinuation were assessed at each visit and graded according to the Division of AIDS toxicity grading table [20]. Plasma HIV-1 RNA was measured at entry and 6 months.

For participants with HIV-1 RNA >1000 at 6 months, genotypic resistance testing was performed on a 6-month plasma sample, and, in Gp2, on a sample obtained at study entry if the participant also had HIV-1 RNA >1000 copies/mL at that time (for those with HIV-1 RNA ≤1000 copies/mL at entry, no resistance testing was performed on the entry sample and any mutations identified during follow-up were considered to be new). Resistance testing was performed in College of American Pathologists–accredited specialty laboratories (BARC-SA and Lancet Laboratories, and Pittsburgh Virology Speciality Laboratory). Drug resistance mutations were identified using the Stanford Algorithm (version 8.8).

Enrollment occurred from October 2019 to July 2022 but was paused from March to July 2020 because of the COVID-19 pandemic. During the pandemic, remote data collection was allowed if a participant was unable to attend a scheduled visit or a site was unable to conduct nonessential visits in the clinic.

## OUTCOMES AND STATISTICAL ANALYSES

### Primary Outcome Measures

The primary virologic outcome was suppression of plasma HIV-1 RNA to ≤1000 copies/mL at 6 months after starting TLD. The proportion of study participants with HIV-1 RNA ≤1000 copies/mL was estimated, together with the associated 2-sided exact 95% CI calculated based on the binomial distribution.

The primary resistance outcome was virologic failure (HIV-1 RNA >1000 copies/mL) with new DTG-associated resistance mutations at 6 months after starting TLD. New DTG resistance mutations were defined as those detected at the time of the failing measurement that were not detected at the time of TLD initiation. Changes in mixture mutations were not counted as new. Within each group, the proportion with HIV-1 RNA >1000 copies/mL and new DTG resistance mutations was estimated, together with the associated 2-sided exact 95% CI.

For the primary outcome, the analysis population included all participants who remained on TLD and received a 6-month HIV-1 RNA measurement. Subgroup comparisons by sex at birth were descriptive for the primary virologic outcome, showing the number and percentage of participants having the outcome of interest by sex at birth within each study group. Predictors of viral suppression at 6 months were analyzed using logistic regression, using the following prespecified variables in univariable models: sex at birth, age at TLD start, baseline CD4 count, and baseline log_10_ HIV-1 RNA.

### Other Outcome Measures

Secondary and exploratory outcomes included suppression of plasma HIV-1 RNA to ≤200 and <50 copies/mL at 6 months, frequency of adverse events leading to TLD discontinuation, and frequency of clinical events relevant to TLD.

### Sample Size

Sample sizes of 360 participants in Gp1 and 180 participants in Gp2 were chosen to address the primary objective of estimating the virologic success rate at 6 months after starting TLD among those still on TLD at 6 months. For Gp1, the width of a 95% CI would be approximately ±3.1% if the success rate was 90% as hypothesized. For Gp2, the width of a 95% CI would be approximately ±5.2% if the success rate was 85% as hypothesized.

## RESULTS

**Figure 1.**
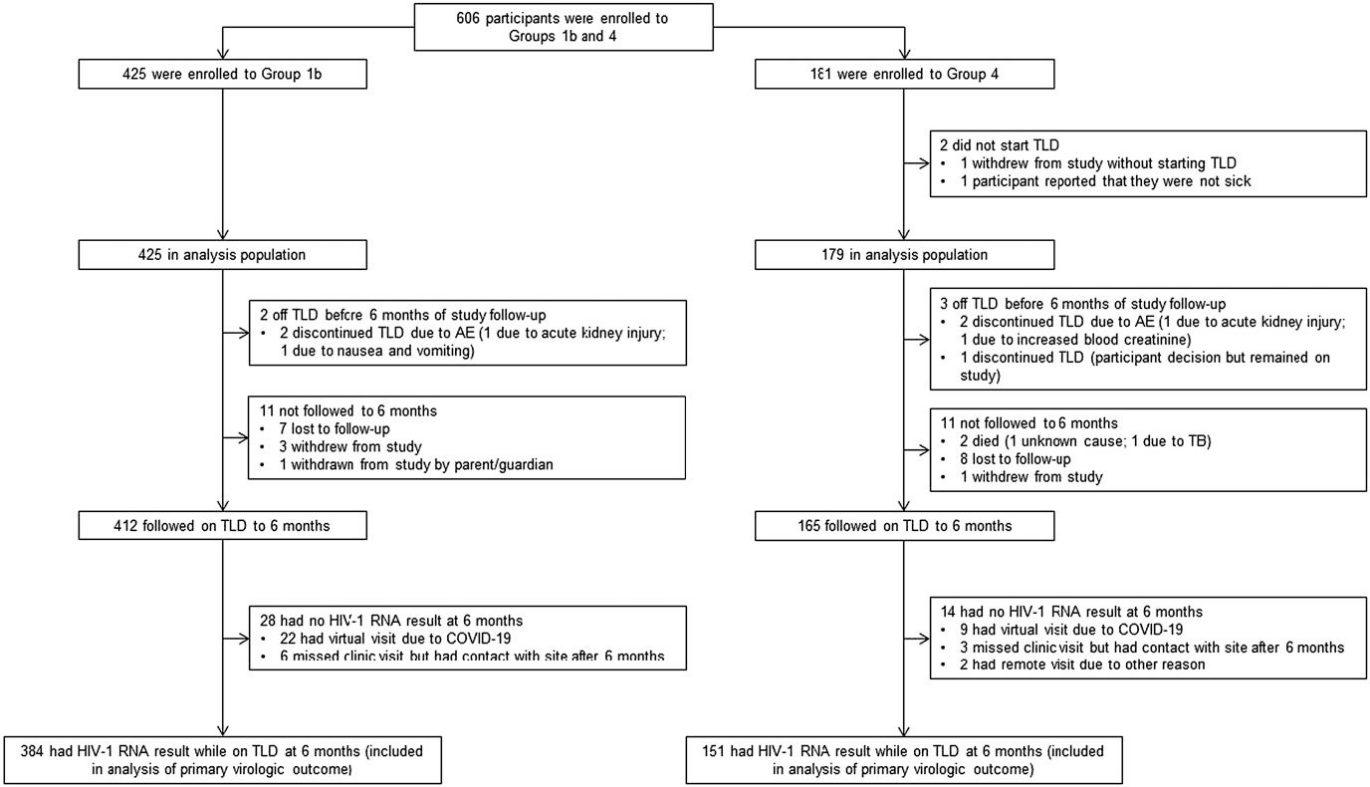
STROBE diagram.

From October 2019 to July 2022, 425 participants were enrolled into Gp1 and 181 participants were enrolled into Gp2 (Figure 1). Two participants in Gp2 did not start TLD, giving an analysis population of 179 participants in Gp2. Participant characteristics are provided for each cohort in Table 1.

**Table 1.** Baseline Characteristics

		Group 1b (N = 425)	Group 4 (N = 179)
Sex	Female	339 (80%)	75 (42%)
Gender identity	Cisgender	425 (100%)	178 (99%)
	Transgender spectrum	0 (0%)	1 (1%)
Age (y)	Median (IQR)	41 (33, 47)	35 (28, 42)
	Min, Max	10, 74	18, 61
Country	Haiti	7 (2%)	47 (26%)
	Kenya	123 (29%)	29 (16%)
	Malawi	40 (9%)	71 (40%)
	South Africa	140 (33%)	4 (2%)
	Uganda	57 (13%)	25 (14%)
	Zimbabwe	58 (14%)	3 (2%)
HIV-1 RNA (copies/mL)	<50	402 (95%)	25 (14%)
	50–200	13 (3%)	1 (1%)
	201–1000	10 (2%)	10 (6%)
	1001–10 000	0 (0%)	30 (17%)
	10 001–100 000	0 (0%)	65 (36%)
	>100 000	0 (0%)	48 (27%)
CD4 count (cells/mm³)	Median (IQR)	676 (477, 893)	302 (165, 517)
	Min, Max	56, 2277	1, 1574
	# missing	10	10

In Group 4, the median HIV-1 RNA was 4.4 log_10_ copies/mL (IQR: 3.5, 5.1).

Abbreviation: IQR, interquartile range.

Two participants (0.5%) in Gp1 and 3 (1.7%) in Gp2 discontinued TLD by 6 months (Figure 1). Two (0.5%) participants in Gp1 and 12 participants (6.7%) in Gp2 experienced at least 1 targeted clinical event through to 6 months. Both participants in Gp1 had diabetes. In Gp2, 7 participants (3.9%) had tuberculosis, 4 (2.2%) had AIDS-defining conditions, 3 (1.7%) died (1 from TB, 1 from acute renal failure, and 1 unknown cause), and 1 (0.6%) each had cancer and immune reconstitution inflammatory syndrome.

Among participants followed on TLD through 6 months, 384 (93.2%) in Gp1 and 151 (91.5%) in Gp2 had a 6-month HIV-1 RNA result and were included in the analysis of the primary outcome measure. Twenty-two participants (5.3%) in Gp1 and 9 participants (5.5%) in Gp2 did not have a result because they had a remote visit during the COVID-19 pandemic where a sample could not be obtained. For the remaining 11 participants (6 [1.5%] in Gp1 and 5 [3.0%] in Gp2) with no result, there was contact after 6 months but no physical study visit at 6 months because of missed or remote visits (considered unrelated to the pandemic).

The proportion of participants with HIV-1 RNA ≤1000 copies/mL was 99% (n = 380) in Gp1 and 90% (n = 136) in Gp2. The exploratory virologic outcomes using thresholds of 200 and 50 copies/mL were achieved in 98% and 96% of participants in Gp1, respectively, and in 87% and 85% of participants in Gp2 (Table 2).

**Table 2.** Proportion of Participants on TLD at 6 Months With HIV-1 RNA Suppressed to 3 Thresholds: ≤ 1000, ≤ 200, and <50 Copies/mL

	Group 1b (N = 425)		Group 4 (N = 179)	
	N/total on TLD with measurement	% (exact 95% CI)	N/total on TLD with measurement	% (exact 95% CI)
Primary virologic outcome:	**…**	**…**	**…**	**…**
HIV-1 RNA ≤1000 copies/mL	380/384	99.0 (97.4–99.7)	136/151	90.1 (84.1–94.3)
Exploratory virologic outcomes:	**…**	**…**	**…**	**…**
HIV-1 RNA ≤200 copies/mL	378/384	98.4 (96.6–99.4)	132/151	87.4 (81.0–92.3)
HIV-1 RNA <50 copies/mL	369/384	96.1 (93.6–97.8)	128/151	84.8 (78.0–90.1)

The total on TLD with measurement was noticeably impacted by the number of participants with missed clinic visits and with virtual visits due to the COVID-19 pandemic at 6 m (see Figure 1).

Abbreviation: TLD, tenofovir/lamivudine/dolutegravir.

All participants who had a missing VL because of virtual visits during the COVID-19 pandemic were from 1 site. A sensitivity analysis that excluded all participants at this site showed similar results (99% in Gp1 and 91% in Gp2 with HIV-1 RNA ≤1000 copies/mL) to the primary analysis. In additional sensitivity analyses, the proportion of participants in Gp1 with HIV-1 RNA ≤1000 copies/mL was 99% (exact 95% CI, 98-100) under the best-case scenario (missing results were imputed as HIV-1 RNA ≤1000 copies/mL) and 95% (exact 95% CI, 92-97) under the worst-case scenario (missing results imputed as HIV-1 RNA >1000 copies/mL). In Gp2, the proportion was 91% (exact 95% CI, 86-95) under the best-case scenario and 83% (exact 95% CI, 76-88) under the worst-case scenario. Note that among the 28 participants in Gp1 and 12 in Gp2 who had no 6-month VL but who were seen in clinic thereafter, all had HIV-1 RNA ≤1000 copies/mL when seen next in-clinic (and all except 1 were <50 copies/mL), so more consistent with the best-case rather than worst-case scenario.

In Grp 1, the proportion of participants with HIV-1 RNA ≤1000 copies/mL at 6 months was 99% in females and 100% in males; in Grp 2, this proportion was 95% for females and 86% for males. Analyses of predictors of viral suppression were not performed in Gp1 because of very high suppression rates. In Gp2, higher age at TLD initiation was associated with greater odds of viral suppression (odds ratio [OR] per 10 years, 4.59; 95% CI, 1.98-10.7) and higher quantitative baseline HIV-1 RNA was associated with lower odds (OR per 1 log_10_ copies/mL higher: 0.56; 95% CI, 0.32-1.00) of HIV-1 RNA ≤1000 copies/mL at 6 months. Sex at birth was not significantly associated with viral suppression (OR, 3.35; 95% CI, 0.90-12.41; *P* = .070).

One of 4 participants in Gp1 who had HIV-1 RNA >1000 copies/mL at 6 months had a mutation (T97A/T) detected, which was presumed to be new, and that was possibly selected by DTG; it is noteworthy that this participant had HIV-1 RNA <40 copies/mL at study entry. The proportion of participants with new resistance in Gp1 was therefore 0.3% (1/383; exact 95% CI, 0-1.5). No new DTG resistance mutations were observed among the 15 participants in Gp2 with HIV-1 RNA >1000 copies/mL at 6 months. The proportion of participants with new resistance in Gp4 was therefore 0% (0/151; exact 95% CI, 0-2.4). Four participants in Gp2 who had DTG resistance mutations (L74IMV, T97AT, G163 K, and E157Q) at 6 months showed similar patterns of resistance at study entry.

## DISCUSSION

We report primary results from an observational cohort of PWH who were receiving ART through the PEPFAR program in Africa and Haiti. This is one of the few studies providing data on early therapeutic efficacy and emergence of HIV drug resistance after switch to TLD for virologically suppressed participants taking NNRTI-based ART and initiation of TLD for ART-naïve participants from implementation program settings outside of a clinical trial context. We found that in an already well-suppressed cohort on NNRTI-based first-line ART (Gp1), 99% maintained plasma HIV-1 RNA suppression (≤1000 copies/mL) at 6 months after switching to TLD. In a parallel cohort of ART-naïve individuals (Gp2), 90% achieved this outcome. The lower rate of viral suppression among ART-naïve individuals is not unexpected: those starting ART may have yet to fully suppress because of shorter duration of ART, or they may be less adherent to a treatment regimen than individuals who have already achieved suppression on a first-line regimen. Rates of viral suppression were slightly lower using thresholds of ≤200 copies/mL and <50 copies/mL. Among participants with viral load >1000 copies/mL at 6 months, development of new DTG resistance at 6 months was low at 0.3% for those switching from NNRTI-based therapy and 0% for those who were ART-naïve.

These results are consistent with the findings of randomized clinical trials that evaluated DTG in switch and ART initiation studies [4, 5, 21, 22]. It is highly encouraging that our results are also consistent with the earlier findings from observational studies in sub-Saharan Africa. A facility-based survey conducted at 2 ART reference treatment centers in Cameroon showed an overall viral suppression rate (<1000 copies/mL) of 96% after 14 months of therapy in individuals either initiating or transitioning to TLD [23]. Another cohort in Lesotho (DO-REAL study) reported on PWH transitioning to a DTG-containing regimen from a NNRTI-based regimen with excellent virologic outcomes (98% had viral load <1000 copies/mL at 16 weeks, with no DTG resistance observed) [24]. In a large cohort from Malawi, 99% of patients with preswitch suppression on first-line ART maintained viral suppression after switching to TLD [25]. In the AFRICOS study, an observational cohort including 12 PEPFAR-supported clinical sites in Nigeria, Kenya, Uganda, and Tanzania, 94% of participants achieved viral suppression (<1000 copies/mL) after switching to TLD [26–28], and 84% of participants maintained a viral load of <50 copies/mL [27].

We noted an unexpectedly high proportion (20%) of participants in Gp2 with HIV-1 RNA <1000 copies/mL before starting TLD, including 14% with HIV-1 RNA <50 copies/mL. This level of HIV-1 RNA suppression could be due to spontaneous HIV-1 control, or from undisclosed use of ART, which has been reported previously, and which may explain the small number of DTG resistance mutations which were present at study entry [29].

Among ART-naïve PWH initiating TLD, there was no development of new resistance to DTG through 6 months, a finding similar to that in pivotal phase 3 studies [1, 4–7, 9, 30]. Four participants had DTG resistance mutations (L74IMV, T97AT, G163K, and I157Q) at 6 months, but had similar patterns of resistance at study entry. Among participants switching from NNRTI-based regimens, only 1 new mutation (T97A/T) was observed. None of these mutations decrease susceptibility to dolutegravir, unless in combination with other INSTI-resistance mutations [31, 32] (35). These results are similar to other studies which reported no or low rates of DTG resistance among patients initiating TLD or switching from suppressive regimens [24, 25, 33]. This finding is particularly salient for implementers and policymakers as they consider scaling up resistance testing versus intensifying efforts to support differentiated adherence counseling. These results support the virological effectiveness of pragmatic transitioning as observed in this study.

This study was conducted at PEPFAR sites that are affiliated with ACTG sites. This may potentially limit the generalizability of our findings. In addition, the follow-up period was relatively short, and a small number of participants discontinued the study or lacked viral load results.

## CONCLUSION

This study demonstrates that TLD is associated with high rates of tolerability and viral suppression in individuals who are ART-naïve or suppressed on first-line NNTRI-based regimens, without emergence of clinically significant DTG resistance mutations after 6 months of treatment.
